# Design and Development of Solid Electrolyte Materials Inspired and Guided by In- depth Crystal Structure Characterizations

**DOI:** 10.1063/4.0000851

**Published:** 2025-10-27

**Authors:** Zhantao Liu, Jue Liu, Yifei Mo, Hailong Chen

**Affiliations:** 1 Georgia Institute of Technology, Atlanta, GA, USA; 2 Oak Ridge National Lab, Oak Ridge, TN, USA; 3 University of Maryland, College Park, MD, USA

## Abstract

The superionic transition (SIC) in solid-state ionic conductors is a critical phenomenon that significantly impacts ionic transport and, consequently, the performance of all-solid-state batteries. A key signature of type II SIC is a change in the slope of the Arrhenius conductivity plot. In our previous study[1], we observed such a slope change in Li-based halide electrolytes and attributed it to grain boundary sintering during hot pressing. This extrinsic effect arises from improved grain connectivity at elevated temperatures, reducing interfacial resistance and leading to an apparent enhancement in ionic conductivity. The findings emphasized the role of processing conditions in shaping transport properties, highlighting grain boundary engineering as a tool to optimize electrolyte performance.

However, recent work[2] reveals that a similar slope change can also stem from an intrinsic origin: the onset of a superionic transition. Using a combination of in situ synchrotron X-ray and neutron diffraction, along with ab initio molecular dynamics simulations, we demonstrate that in Li3YCl6 and related halides, the transition is driven by the emergence of collective anion motion, which fundamentally alters the energy landscape for Li ion diffusion. This intrinsic mechanism is distinct from grain boundary effects and represents a true transition into a dynamically disordered state with exceptionally low activation energy for ion transport.

By reconciling these extrinsic and intrinsic mechanisms, our work provides a comprehensive understanding of superionic transitions in solid electrolytes. These insights not only clarify discrepancies between experimental and theoretical conductivity values but also offer new design principles for engineering next- generation solid electrolytes with tunable transport properties. Leveraging the newly revealed insights, two groups of new halide solid electrolytes were designed and very high room temperature ionic conductivities of 7 mS/cm and 12 mS/cm, respectively, were successfully achieved, which further demonstrating that understanding the interplay between structural disorder, anion dynamics, and processing- induced modifications will be crucial for advancing high-performance solid-state batteries.

## References

[1] Z.T. Liu, S. Ma, J. Liu, S. Xiong, Y.F. Ma, H.L. Chen, High Ionic Conductivity Achieved in Li3Y(Br3Cl3) Mixed Halide Solid Electrolyte via Promoted Diffusion Pathways and Enhanced Grain Boundary, Acs Energy Letters, 6 (2021) 298-304. 10.1021/acsenergylett.0c01690

[2] Z.T. Liu, P.H. Chien, S. Wang, S.W. Song, M. Lu, S. Chen, S.M. Xia, J. Liu, Y.F. Mo, H.L. Chen, Tuning collective anion motion enables superionic conductivity in solid-state halide electrolytes, Nature Chemistry, 16 (2024). 10.1038/s41557-024-01634-6 39313631

